# Toward a quality-managed operational architecture for wastewater surveillance of zoonotic and emerging pathogens

**DOI:** 10.1128/aem.00950-26

**Published:** 2026-07-10

**Authors:** Rajendra Kothavade

**Affiliations:** 1 Horsham Water and Sewer Authority, Horsham, Pennsylvania, USA; 2 Rhode Island Department of Health, Environmental Science Center, Providence, Rhode Island, USA; 3 PFAS and Emerging Contaminants, Water Environment Federation (WEF), Alexandria, Virginia, USA; The Pennsylvania State University, University Park, Pennsylvania, USA

**Keywords:** wastewater-based epidemiology, zoonotic pathogens, emerging infectious diseases, quality management systems, RT-qPCR, digital PCR, wastewater surveillance, public health monitoring, sequencing

## Abstract

Wastewater surveillance provides population-level insights into infectious disease dynamics independent of clinical testing, with increasing relevance for zoonotic and emerging pathogens, such as avian influenza A (H5 subtypes), influenza B, respiratory syncytial virus, measles, and mpox. This minireview integrates quality management system (QMS) principles across the entire wastewater surveillance workflow, including sample collection, laboratory analysis, data interpretation, and reporting, to ensure reliability, reproducibility, and cross-site comparability. The review presents the novel framework of wastewater surveillance as a structured operational architecture, highlighting how biological variability, sampling design, analytical workflows, and interpretive strategies interact within a unified system. It provides decision-oriented guidance for interpreting heterogeneous and low-prevalence signals, integrating system-level metadata, tiered analytical approaches (reverse transcription quantitative PCR [RT-qPCR], digital PCR, sequencing), and strategies for linking molecular measurements to actionable public health decisions. Additionally, it addresses the incorporation of One Health perspectives and multi-level governance—from municipal infrastructure to national coordination—to enhance early detection, outbreak mitigation, and coordinated response. By translating complex environmental and molecular inputs into a coherent operational framework, this work equips public health agencies and researchers to design resilient, scalable, and evidence-based wastewater surveillance systems that support early warning and effective management of emerging infectious disease threats.

## INTRODUCTION

Wastewater-based epidemiology has emerged as a complementary approach to conventional surveillance, providing population-level insights into infectious disease dynamics by analyzing communal wastewater systems. Its utility has been demonstrated during the COVID-19 pandemic, where wastewater signals enabled early detection of community transmission and supported public health decision-making. Beyond SARS-CoV-2, wastewater surveillance holds significant potential for monitoring zoonotic and emerging infectious diseases, including respiratory viruses, such as influenza A viruses (particularly zoonotic subtypes such as H5N1), influenza B virus, and respiratory syncytial virus, as well as re-emerging zoonotic pathogens, such as measles virus and mpox virus, for which timely detection remains a persistent global challenge (1–7).

Zoonotic and emerging pathogens present distinct surveillance complexities arising from heterogeneous transmission pathways, multi-host reservoirs, and variable shedding dynamics (8). In contrast to traditional clinical surveillance, which relies on case detection within defined populations, wastewater systems integrate signals from diverse sources, including human excretion, animal waste inputs, and environmental contamination (9, 10). Consequently, wastewater-derived measurements represent composite system outputs rather than direct indicators of infection prevalence, requiring careful interpretation within a biological and environmental context.

The reliability of wastewater surveillance is further influenced by system-level factors, including hydraulic variability, environmental degradation of target analytes, and heterogeneity in sampling design and analytical workflows. These sources of variability are amplified in the context of emerging and low-prevalence pathogens, where signals may approach detection limits and are more susceptible to methodological inconsistencies (11, 12). Without structured control across these domains, wastewater data may be difficult to compare across sites and over time, limiting its utility for public health decision-making.

Despite rapid advances in molecular detection technologies and increasing global adoption of wastewater surveillance, implementation remains fragmented, with variability in sampling strategies, analytical methods, data normalization approaches, and interpretive frameworks. Existing efforts often emphasize individual components of the surveillance process without addressing their interdependence within a unified operational system. This fragmentation poses a barrier to scalability, comparability, and integration into routine public health infrastructure (12–14).

Addressing these challenges requires a system-level perspective that integrates biological understanding, sampling design, analytical workflows, and data interpretation within a structured operational framework (15). Quality management system (QMS) principles provide a foundation for such integration by establishing standardized processes, performance monitoring, and continuous improvement mechanisms across the surveillance continuum (16). While QMS approaches are well established in laboratory settings, their application to wastewater surveillance as an end-to-end system remains underdeveloped.

This review synthesizes current knowledge on the biological and environmental determinants of wastewater signals, examines pre-analytical and analytical sources of variability, and proposes a QMS-governed operational architecture for wastewater surveillance of zoonotic and emerging infectious diseases. By framing wastewater surveillance as an integrated system rather than a collection of independent methodologies, the work aims to support the development of reliable, comparable, and actionable surveillance platforms for public health applications.

Several recent reviews have examined wastewater-based epidemiology in the context of infectious disease surveillance, including methodological standardization and reporting frameworks (17), advances in sequencing and genomic surveillance (18), variability in sampling design (19), and integration with public health systems (20). These studies have provided important insights into specific components of surveillance, including sampling strategies, molecular detection platforms, sequencing approaches, and the epidemiologic interpretation of wastewater-derived signals. However, these contributions have largely addressed these elements in isolation rather than as interconnected components of a unified operational system. In particular, prior reviews have not systematically examined how biological variability, sampling design, analytical workflows, and interpretive frameworks interact to influence overall system performance, comparability, and decision-making.

In contrast, this review advances a systems-level perspective by explicitly framing wastewater surveillance as a quality-managed operational architecture. By integrating QMS principles across all stages from sampling through interpretation and governance, this work provides a structured framework for reducing variability, improving cross-site comparability, and enabling consistent decision-making. This integrative approach is particularly relevant for zoonotic and emerging pathogens, where low prevalence, heterogeneous inputs, and uncertainty in transmission dynamics underscore the need for standardized, coordinated surveillance systems.

**Fig 1:**
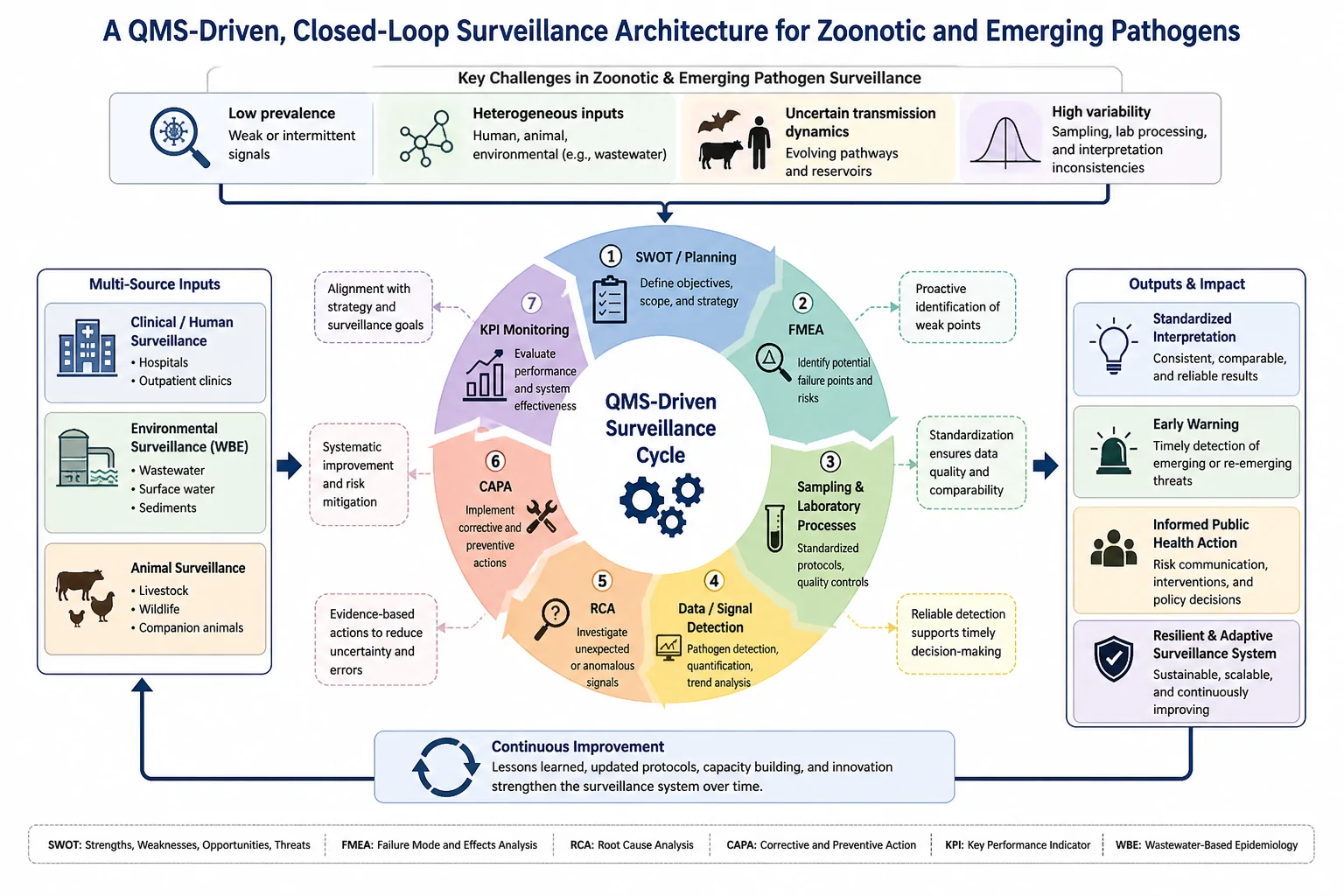
General workflow of wastewater surveillance systems for zoonotic and emerging pathogens, illustrating major stages, including sampling, laboratory analysis, data interpretation, and public health reporting. The framework highlights the integration of environmental, analytical, and epidemiologic information required for surveillance interpretation and response.

Building on these foundations, a systems-level perspective is advanced by explicitly framing wastewater surveillance as a quality-managed operational architecture. Integration of QMS principles across all stages, from sampling through interpretation and governance, provides a structured framework for reducing variability, improving cross-site comparability, and enabling consistent decision-making. This integrative approach is particularly relevant for zoonotic and emerging pathogens, where low prevalence, heterogeneous inputs, and uncertainty in transmission dynamics underscore the need for standardized, coordinated surveillance systems. The proposed operational architecture is illustrated in Fig. 1.

## WASTEWATER SIGNALS IN ZOONOTIC AND EMERGING DISEASE CONTEXTS

Wastewater surveillance signals arise from complex and variable biological processes that are further shaped by environmental transport and system-level dynamics. These factors are particularly pronounced for zoonotic and emerging pathogens, where multiple host species, heterogeneous transmission pathways, and non-human environmental inputs contribute to the composite signals detected in wastewater systems (21–23).

Pathogen shedding represents the primary biological input into wastewater; however, shedding dynamics vary substantially across pathogens, host species, and infection stages. Human infections contribute viral and microbial material through feces, urine, respiratory secretions, and other excreta, while animal reservoirs, including livestock and wildlife, may introduce additional inputs through agricultural runoff, manure management systems, or environmental contamination. For zoonotic pathogens, such as avian influenza viruses, wastewater signals may therefore reflect combined contributions from human and animal sources, complicating attribution and interpretation (24–29).

Current evidence indicates that wastewater surveillance is not limited to enteric pathogens or SARS-CoV-2 but can support monitoring of pathogens with zoonotic or pandemic potential when shedding routes, assay sensitivity, and sampling context are aligned (30). Prototype examples include SARS-CoV-2, mpox virus, and Zika virus, for which human shedding and wastewater detection data support the feasibility of community-level surveillance across pathogens with different transmission routes (30–32). In zoonotic contexts, wastewater signals may arise from human infections, animal reservoirs, or environmental inputs; therefore, detection does not necessarily imply human transmission unless supported by epidemiologic, genomic, and source-associated data (30, 33). Viral sequence information and host-associated biomarkers may provide supportive evidence for distinguishing potential sources of wastewater signals; however, reliable attribution to specific host populations remains challenging, particularly for pathogens with multiple reservoirs or segmented genomes, such as influenza viruses. Although active areas of research include genomic resolution, source-tracking markers, and integrative surveillance approaches, these methods currently provide probabilistic rather than definitive attribution (34, 35). This limitation highlights the need for cautious interpretation of wastewater signals and underscores the importance of integrating wastewater data with complementary epidemiologic, veterinary, and environmental surveillance systems.

These attribution challenges are reflected in pathogen-specific differences in shedding pathways and environmental entry routes. Zoonotic pathogens introduce additional complexity because wastewater signals may arise from multiple host and environmental sources. Avian influenza A viruses, including H5 subtypes, may enter wastewater systems through human infection, livestock-associated waste streams, wild-bird contamination, or agricultural runoff (36, 37). Mpox virus may be detected through shedding from skin lesions, feces, urine, or other bodily fluids during outbreak or low-incidence periods (38, 39), whereas Zika virus and other arboviruses may contribute wastewater signals through urine or other excreta during community transmission (30, 40). Re-emerging pathogens, such as measles virus, further illustrate that wastewater detectability depends on secondary shedding routes, including urine, as well as outbreak scale, sampling frequency, and analytical sensitivity (41, 42). These differences underscore that pathogen-specific shedding biology and environmental entry routes must be considered when interpreting wastewater detections.

An additional interpretive consideration arises for vaccine-preventable diseases. Detection of measles virus RNA in wastewater may reflect both wild-type infections and vaccine-associated shedding, particularly in settings with recent vaccination activity (43, 44). This distinction has important implications for interpretation, as vaccine-derived signals may not indicate active transmission. Differentiation between wild-type and vaccine-derived strains, therefore, requires sequence-based or molecular approaches to support accurate surveillance interpretation.

More broadly, variability in pathogen shedding further complicates the interpretation of wastewater signals across pathogens and contexts. Shedding magnitude and duration are not constant and may differ between symptomatic and asymptomatic infections, across age groups, and between host species. Respiratory viruses, including influenza viruses and respiratory syncytial virus, may exhibit variable or intermittent gastrointestinal shedding, resulting in inconsistent wastewater detectability (45, 46). Similarly, re-emerging pathogens such as measles virus, while primarily transmitted via respiratory routes, may contribute detectable genetic material via secondary shedding pathways during outbreaks (47–49). These variations introduce uncertainty in the relationship between infection prevalence and measured wastewater concentrations.

In addition to variability in biological inputs, wastewater signals are further shaped by environmental transport and transformation processes within sewer systems. Once introduced, pathogen genetic material undergoes dilution, adsorption to solids, degradation, and transport through sewer networks. Hydraulic conditions, including flow variability, residence time, and infiltration and inflow, can independently alter measured concentrations. Viral RNA and DNA may preferentially associate with suspended solids, enhancing detectability in settled solids relative to liquid influent, while also introducing matrix-dependent variability. Environmental factors, such as temperature, chemical composition, and microbial activity, further influence nucleic acid persistence and recovery efficiency (50–53).

To account for these environmental influences, structured approaches are required to quantify and integrate system-level variability into analytical workflows. This can be achieved by incorporating auxiliary system-level data into analysis and interpretation frameworks. For example, flow measurements may be used to normalize pathogen concentrations to flow-adjusted loads, such as genome copies per day, enabling differentiation between true changes in shedding and dilution-driven variability. Similarly, partitioning between liquid and solid fractions can be evaluated through parallel analysis of influent and settled solids, improving detection sensitivity and interpretability. Environmental parameters, such as temperature and residence time, can be incorporated into interpretive frameworks through stratified analyses or adjustment models, enabling more consistent comparisons across sampling events and sites.

By incorporating environmental influences as quantifiable inputs rather than treating them as uncontrolled sources of uncertainty, wastewater surveillance systems can improve the reliability and interpretability of measured signals. This approach is essential for providing public health authorities with consistent and actionable insights for infectious disease monitoring and early warning.

Collectively, these interacting biological and environmental processes result in wastewater measurements that represent emergent system-level signals rather than direct indicators of infection burden. Measured concentrations reflect the combined effects of heterogeneous shedding, multiple-source inputs, environmental transformation, and analytical recovery (24, 54–56) and therefore cannot be interpreted in isolation without consideration of the system context.

This inherent complexity becomes particularly pronounced in the context of zoonotic and emerging pathogens. In these settings, low prevalence, variable shedding pathways, and mixed-source contributions increase uncertainty and reduce signal stability (54, 57, 58). As a result, wastewater surveillance requires structured approaches that account for system-level variability and ensure that observed signals can be interpreted consistently across time and location.

Addressing the complexities inherent in system-level operations goes beyond merely documenting environmental and operational parameters; it demands a strategic integration into analytical and interpretative workflows. To achieve this effectively, a methodical, staged approach is essential. Initially, critical parameters, such as flow rate, precipitation events, temperature, and sampling metadata, must be systematically quantified and recorded using standardized formats. Next, these parameters should be utilized in analytical normalization strategies, transforming concentrations into flow-adjusted loads or categorizing by sample matrix. Subsequently, this normalized data must be assessed against historical baselines under similar environmental conditions to enhance signal stability and interpretability. Ultimately, these cohesive data streams feed into clear decision-making criteria, including thresholds for detecting anomalies, escalating signals, or conducting targeted follow-up sampling. By implementing this framework, system-level variability is not merely documented but actively integrated into the interpretation and decision-making processes within wastewater surveillance systems. This proactive approach ensures consistent interpretation across diverse sites and significantly strengthens the reliability of public health signals derived from wastewater analysis.

To effectively implement these frameworks in practice, it is essential to establish coordinated operational procedures throughout the various stages of the surveillance workflow. Standard operational approaches in wastewater surveillance can greatly benefit from the integration of well-defined protocols. This includes the use of standardized sampling methods with clear criteria for frequency and site selection, ensuring consistent data collection of important metadata, such as flow rates, environmental conditions, and the context of each sample.

Moreover, adopting validated analytical workflows with documented performance characteristics can enhance the reliability of the results. At the interpretive stage, it is valuable to establish predefined baseline definitions, detection thresholds for anomalies, and criteria for signal escalation or targeted follow-up sampling. By incorporating these components into centralized or coordinated reporting systems, comparability across different sites and time periods can be improved. This would enable wastewater surveillance data to be transformed into cohesive, actionable insights for public health, ultimately promoting better community health outcomes.

To effectively apply operational approaches in wastewater surveillance, it is essential to clearly define key sources of variability that influence the interpretation of measured signals. Hydraulic variability involves fluctuations in wastewater flow and composition driven by diurnal patterns, precipitation events, and sewer system dynamics, which can either dilute or concentrate target signals. Sampling heterogeneity reflects the spatial and temporal differences in wastewater composition resulting from an uneven distribution of contributing populations and intermittent shedding. Additionally, analytical workflows encompass laboratory processes, including sample concentration, nucleic acid extraction, and molecular detection, all of which affect assay sensitivity, precision, and reproducibility. A clear understanding of these concepts is vital for recognizing how variability affects wastewater surveillance systems and for developing effective quality management strategies.

The necessity of robust integrated approaches is highlighted by the recent outbreak dynamics in the United States. These outbreaks reveal the shifting landscape of zoonotic and emerging pathogens and their significant relevance to wastewater surveillance (59–62). The 2022 mpox outbreak, for instance, demonstrated swift nationwide spread, while ongoing reports indicate the emergence of new clades with localized transmission. Similarly, the continual genomic evolution of SARS-CoV-2 has produced variants that alter transmission patterns. Moreover, measles has resurfaced as an urgent public health threat, with outbreaks occurring in highly interconnected communities where early detection is crucial (63–66). These examples underscore the urgent need for surveillance systems capable of identifying both established and emerging pathogens across diverse transmission scenarios. In this context, wastewater surveillance, when supported by structured sampling, comprehensive analytical workflows, and quality management principles, can significantly improve early detection, bolster situational awareness, and provide vital information for timely public health responses.

## SAMPLING DESIGN AS A CONTROLLED SYSTEM IN ZOONOTIC AND EMERGING SURVEILLANCE

The reliability of interpreting wastewater surveillance data hinges on the implementation of effective sampling strategies that can accurately capture representative signals from the broader system. These strategies must account for the inherent biological variability and the complex hydraulic conditions that affect wastewater flow and composition. As highlighted in previous studies (22, 67, 68), wastewater measurements provide a comprehensive overview of inputs influenced by the various dynamic processes discussed in “Wastewater signals in zoonotic and emerging disease contexts,” above. Consequently, the design of the sampling approach is crucial. It acts as the primary mechanism to minimize pre-analytical variability, ensuring that samples collected truly represent the conditions of the wastewater system at the time of sampling. By establishing consistent and representative sampling conditions, it becomes possible to better interpret the downstream analytical results with meaning and significance (67–71). This approach not only enhances data reliability but also supports informed decision-making by providing analytical outcomes from the sampled wastewater.

Selection of sampling strategies should be guided by surveillance objectives and epidemiologic context (14, 69). For routine population-level monitoring, composite sampling at wastewater treatment plant influent provides integrated signals representative of large catchment populations and supports longitudinal trend analysis. In contrast, targeted surveillance for zoonotic or emerging pathogens, particularly in low-prevalence or early-detection scenarios, may benefit from upstream or near-source sampling, such as manholes, sewer interceptors, or facilities associated with high-risk populations or animal–human interfaces. Sampling frequency should also be adjusted to surveillance goals, with higher frequency during periods of suspected emergence or outbreak and lower frequency during stable baseline conditions (12).

In wastewater-based epidemiology, pathogens shed by infected individuals enter sewer systems after flushing in households, healthcare facilities, and community settings. This material mixes with wastewater as it travels through sewer networks and converges at collection points, such as manholes or influents at treatment plants. Composite samples collected with autosamplers are preferred for surveillance, as they integrate inputs over time and capture variability in shedding patterns and flow conditions, thus providing a representative measure of community infection dynamics (72, 73). In contrast, grab samples reflect conditions at a single time point and may miss intermittent shedding events (14). The choice between grab and composite sampling involves a trade-off between temporal resolution and representativeness, with composite sampling generally favored for trend analysis and population-level surveillance. Grab sampling is typically used for rapid assessments when necessary. These considerations demonstrate that sampling design is context-dependent in wastewater surveillance systems.

Sampling location is a critical determinant of representativeness and must be aligned with surveillance objectives. In municipal systems, influent sampling at wastewater treatment plants provides integrated, population-level signals across entire sewersheds, supporting broad community surveillance. In contrast, upstream or targeted sampling locations can improve spatial resolution and enable focused monitoring of specific populations or risk environments (74–76). For zoonotic and emerging pathogens, such targeted approaches are particularly important where transmission is linked to defined interfaces between human, animal, and environmental systems (77). Surveillance for mpox may prioritize catchments with high international mobility, such as areas surrounding major transportation hubs, whereas monitoring for avian influenza A (H5N1) may include wastewater systems serving agricultural regions, dairy operations, or sewer lines proximal to livestock facilities (61, 78, 79). Similarly, for re-emerging pathogens, such as measles, where transmission may occur at low levels prior to clinical recognition, wastewater surveillance can provide an early indication of reintroduction when sampling strategies are aligned with high-risk or highly connected populations (60, 80). These context-specific strategies illustrate that sampling design for zoonotic and emerging surveillance must be guided by epidemiologic risk rather than uniform, infrastructure-based approaches.

Sampling frequency influences both sensitivity and temporal resolution. Higher-frequency sampling enhances the ability to detect rapid changes in pathogen circulation, particularly during early outbreak phases or in low-prevalence conditions. However, optimal frequency is system-dependent and should reflect epidemiologic risk, hydraulic variability, and operational capacity. Composite sampling, particularly when flow-weighted, improves representativeness by integrating wastewater signals over time and reducing short-term variability associated with intermittent shedding and diurnal flow patterns (81). Representative sampling considerations for selected zoonotic and emerging pathogens are summarized in Table 1.

**TABLE 1:** Decision-oriented deployment of molecular platforms for wastewater surveillance of zoonotic and emerging pathogens^*a*^

Pathogen/target	Surveillance objective	Analytical context	Platform selection rationale	Recommended deployment strategy
Influenza A (M gene)	Population-level trend monitoring	Moderate to high prevalence; seasonal circulation	High-throughput, scalable detection sufficient for trend analysis	RT-qPCR or dPCR deployed based on system design; dPCR may be used for enhanced resolution during low-prevalence or resurgence phases
H5 (HA subtype)	Early detection of zoonotic emergence	Low prevalence; subtype-specific signal	Increased analytical sensitivity required for early detection of rare subtype signals	RT-qPCR or dPCR applied depending on sensitivity requirements; dPCR provides enhanced detection confidence under low-abundance conditions
Influenza B (NS gene)	Seasonal transition monitoring	Declining or low-prevalence conditions	Improved resolution beneficial for detecting low-level persistence	RT-qPCR or dPCR deployed based on analytical needs; dPCR supports improved detection confidence at low concentrations
SARS-CoV-2 (N1/N2)	Longitudinal trend analysis and resurgence detection	Variable prevalence; potential assay discordance	Precision and improved tolerance to inhibition beneficial in complex wastewater matrices	RT-qPCR or dPCR applied depending on surveillance objectives; dPCR may enhance robustness and quantitative confidence
RSV (N gene)	Early seasonal emergence detection	Low initial prevalence; increasing transmission	Enhanced sensitivity supports early detection under low-abundance conditions	Flexible deployment of RT-qPCR or dPCR based on epidemiologic phase and sensitivity requirements
mpox (F3L gene)	Outbreak detection and inter-outbreak surveillance	Low-incidence or re-emergence conditions	High sensitivity improves detection under low-signal conditions	RT-qPCR or dPCR applied depending on detection requirements; dPCR supports low-level signal detection
Measles (N gene)	Detection of reintroduction in elimination settings	Very low prevalence; sporadic shedding	High analytical sensitivity required for early detection of reintroduction events	RT-qPCR or dPCR deployed based on sensitivity needs; dPCR enhances detection confidence under low-prevalence conditions

^*a*^ Molecular platform selection is aligned with surveillance objectives, analytical context, and detection requirements across representative targets (82–104). RT-qPCR and dPCR may be deployed as primary or complementary analytical platforms depending on surveillance design, resource availability, and detection requirements.

Hydraulic conditions introduce additional variability that must be explicitly incorporated into the sampling design. Infiltration and inflow, storm events, and fluctuations in wastewater flow can dilute, concentrate, or temporally shift measured signals independent of underlying transmission dynamics (105). Failure to account for these factors may lead to misinterpretation of apparent trends. Accordingly, sampling strategies should incorporate flow-aware approaches and be accompanied by documentation of environmental and operational conditions during sample collection. These effects are best addressed through longitudinal interpretation within a site-specific hydraulic context rather than reliance on isolated concentration values.

Using solid-based sampling can significantly improve detection sensitivity, especially in low-prevalence settings (106). However, it is essential to standardize testing protocols, sample collection techniques, and processing methods to minimize variability (107). The choice of sample matrix should be carefully aligned with specific surveillance objectives—such as the types of pathogens being monitored or the populations at risk and with the laboratory’s capacity in terms of resources, technology, and expertise. Establishing a coordinated framework is crucial, as it facilitates collaboration among stakeholders to create common guidelines and best practices. This clarity in purpose and process will enhance the reliability of findings and ensure data comparability across different studies and settings.

The design of sampling is critical as it directly impacts downstream measurements, making it essential to support it with structured metadata and standardized operational practices. Thorough documentation of sampling locations, composite methodologies, flow conditions, matrix types, and sample handling procedures is vital for accurately interpreting wastewater signals and identifying genuine epidemiological trends, rather than misattributed sampling artifacts (108). Without such standardization, the variability introduced during sampling can propagate through analytical workflows, jeopardizing cross-site comparability. It is crucial to address operational and system-level factors that affect sampling representativeness and to implement robust quality management system controls, as summarized in Table 2.

**TABLE 2:** Operational and system-level determinants of wastewater surveillance representativeness and corresponding QMS controls (14, 69, 109–123)

Technical impact	Issue	Surveillance risk	QMS control/mitigation	Required metadata
Dilution of target signal; altered solids concentration; increased hydraulic variability	Infiltration and inflow (I/I) from groundwater or stormwater	False low concentrations; misinterpretation of declining trends during rainfall	Use flow-weighted composite sampling when feasible; document rainfall and influent flow; interpret trends longitudinally rather than through isolated cross-site comparisons	Rainfall indicators; influent flow; sewer type (combined or separate); known I/I conditions
Rapid flow fluctuations; altered diurnal input patterns	Storm events and wet-weather flow spikes	Reduced representativeness of time-based composites	Increase aliquot frequency during high variability; prioritize flow-weighted sampling when possible	Composite type; aliquot interval; flow during sampling window
Non-uniform flow depth; sediment accumulation; intermittent flow	Manhole or upstream autosampler placement	Bias toward settled solids or low-flow fractions	Select hydraulically stable locations; avoid backwater zones; verify intake depth; perform periodic field validation	Site ID; flow depth; intake position; site description
Blending of subcatchments; industrial discharge pulses	Wastewater treatment plant influent variability	Smoothing of localized outbreak signals	Supplement plant influent sampling with targeted upstream sampling when warranted	Plant flow; catchment coverage; industrial discharge notes
Over- or under-representation during high- or low-flow periods	Time-based composite sampling	Bias during strong diurnal or storm-driven variation	Prefer flow-weighted composites where possible; shorten aliquot intervals when needed	Composite method; interval timing; total volume
Insufficient material for replicate testing and archiving	Inadequate composite volume	Reduced QA/QC reliability; limited confirmatory or retrospective testing	Define minimum composite volume to support replicate molecular testing and archiving	Total composite volume; subsample volumes
Uneven distribution of solids-associated targets	Poor homogenization prior to subsampling	High variability between replicate aliquots	Standardize mixing method and duration; subsample immediately after mixing	Mixing method; mixing duration; time between mixing and aliquoting
Uneven distribution of particulate-associated targets	Settling during dispensing	Underestimation or inconsistent quantification	Maintain agitation during dispensing; remix before each aliquot when needed	Dispensing protocol; agitation method
RNA degradation; reduced detection sensitivity	Delayed transport or improper storage	False negatives or low quantification	Maintain cold chain; define maximum hold time; archive aliquots at appropriate cryogenic temperature when feasible	Collection time; storage temperature; time to extraction
Limited interpretability of anomalous trends	Inconsistent metadata documentation	Reduced scientific defensibility and cross-site comparability	Require standardized metadata reporting for each sampling event	Sampling location; composite type; matrix; environmental conditions; QA/QC metrics
Limited contextualization of wastewater signals due to incomplete system-level data	Limited availability of system-level metadata (e.g., flow, environmental conditions, infrastructure data)	Reduced interpretive confidence; increased uncertainty in distinguishing true signal changes from system variability	Where direct measurement is not feasible, use proxy indicators or centralized data sources (e.g., treatment plant records, meteorological data); explicitly document data availability limitations	Source of metadata; data availability notes; proxy indicators used

Sampling is an active control measure in wastewater surveillance, not merely a passive step. For zoonotic and emerging pathogens, where signals may be weak and variable, a structured sampling design and standardized documentation are essential. This ensures that measurements accurately reflect true transmission dynamics rather than random system fluctuations.

## ANALYTICAL ARCHITECTURE AND QMS INTEGRATION IN WASTEWATER SURVEILLANCE

In wastewater surveillance, implementing a structured QMS is essential to ensure consistent, reliable, and comparable data across all stages of the monitoring process. By embedding QMS principles throughout the surveillance continuum, system performance can be systematically strengthened from data generation to decision-making. At the sampling stage, the adoption of standardized protocols and clearly defined site selection criteria ensures that collected samples are representative, traceable, and interpretable (16, 124). In laboratory analysis, robust quality controls—including extraction controls, inhibition assessments, and method validation procedures—enhance analytical accuracy and reproducibility (125). During data interpretation, the use of predefined thresholds, normalization strategies, and context-aware decision frameworks supports consistent and reliable evaluation of pathogen signals (126). At the reporting stage, structured data formats and centralized governance mechanisms facilitate cross-site comparability, enable longitudinal analyses, and promote coordinated learning across surveillance systems (125).

By integrating these practices, wastewater surveillance systems can more effectively translate heterogeneous molecular, environmental, and epidemiologic inputs into actionable public health intelligence. The implementation of a well-designed QMS strengthens the overall operational framework by improving measurement reliability, supporting evidence-based decision-making, and enhancing long-term system resilience.

The QMS not only functions as a quality assurance mechanism but also serves as an operational control system within wastewater surveillance. When deviations from established baselines or predefined thresholds are detected, QMS-guided escalation actions may include increasing sampling frequency, conducting targeted upstream investigations, or deploying higher-sensitivity analytical methods. Through this structured and adaptive approach, wastewater surveillance systems can progress from descriptive monitoring toward standardized, decision-support frameworks. Collectively, these interconnected elements establish the quality-managed operational framework depicted in Fig. 1.

Within this framework, the reliability of downstream interpretation depends on the performance and standardization of analytical workflows. While representative sampling establishes the conditions under which wastewater signals can be captured, translating these signals into interpretable data depends on the consistency and robustness of downstream analytical processes. Wastewater represents a complex environmental matrix characterized by variable nucleic acid recovery, matrix-associated inhibition, solids partitioning, and heterogeneous target distribution (107, 115, 127, 128). These factors introduce analytical variability that can obscure true transmission dynamics (69) if not systematically controlled.

To address this variability, molecular detection strategies must be aligned with clearly defined surveillance objectives. In wastewater surveillance, molecular detection typically serves three primary functions: presence–absence detection, quantitative trend monitoring, and genomic characterization. Alignment between analytical objectives and methodological approaches is therefore essential, with key analytical considerations summarized in Table 1.

RT-qPCR remains the primary platform for routine detection and longitudinal trend analysis because of its scalability, sensitivity, and operational feasibility across large surveillance networks (129–131). However, RT-qPCR-based quantification is influenced by amplification efficiency, matrix inhibition, and dependence on calibration curves, which may introduce variability, particularly at low target concentrations (131).

Digital PCR (dPCR) provides complementary analytical capabilities under specific surveillance conditions. Through partition-based measurement, dPCR enables absolute target quantification without dependence on external standards (131–133). In wastewater matrices, dPCR may improve confidence in low-abundance detection and demonstrate greater tolerance to inhibitory effects, making it particularly valuable during early outbreak phases, low-prevalence conditions, or resurgence events in which signals approach analytical limits. Despite these advantages, higher cost, lower throughput, and technical complexity currently constrain its routine deployment as a primary surveillance platform. Importantly, dPCR should not be interpreted as a confirmatory method in the classical diagnostic sense, where confirmation is typically achieved through sequencing or other orthogonal approaches (13). Instead, dPCR may be selectively integrated alongside RT-qPCR depending on surveillance objectives and operational constraints.

In addition to analytical performance, turnaround time remains a critical factor influencing platform selection. RT-qPCR typically offers rapid processing and high throughput, supporting near-real-time monitoring, whereas dPCR may provide enhanced sensitivity with comparable throughput depending on platform configuration (134). Sequencing, while essential for confirmatory analysis and genomic characterization, generally involves longer processing times (135) and is therefore applied selectively according to predefined surveillance priorities. These trade-offs underscore the importance of aligning analytical strategies with both surveillance objectives and timeliness requirements.

Sequencing further extends analytical capability by enabling genomic characterization, variant identification, and detection of emerging or unusual strains (136, 137). In wastewater surveillance, sequencing is most effectively deployed as an escalation strategy triggered by predefined analytical or epidemiologic criteria, such as sustained increases in viral concentration or discordance between assay targets. Targeted amplicon sequencing provides efficiency when specific pathogens are known, whereas untargeted approaches—including metagenomic sequencing and hybrid-capture methods—enable broader detection of known and unknown agents at the expense of increased complexity and resource requirements (136–138). Although currently more common in research and exploratory settings, these approaches are expected to assume an increasingly important role as sequencing workflows, bioinformatic pipelines, and interpretive frameworks mature.

When integrated within a structured, tiered framework, these analytical methods function most effectively as complementary components of a coordinated surveillance architecture. In this hierarchical approach, RT-qPCR supports routine monitoring, dPCR provides enhanced sensitivity and quantitative reliability in analytically challenging scenarios, and sequencing is selectively applied for confirmatory and genomic analyses. This tiered structure aligns analytical intensity with surveillance objectives while maintaining scalability and operational feasibility across surveillance systems.

Within this integrated analytical framework, QMS principles provide the structure necessary to ensure consistency, comparability, and reliability across laboratories and jurisdictions. QMS implementation includes validation of extraction and detection methods, use of internal controls for inhibition and recovery assessment, verification of detection limits, and standardized reporting of molecular targets and quantification metrics (16). Harmonization of target gene selection, including consistent use of conserved genomic regions across platforms, further supports interoperability and comparability across surveillance systems.

A well-designed QMS also strengthens analytical interpretation by integrating environmental and operational metadata, including flow dynamics, temperature, hydraulic conditions, and matrix composition. Standardized metadata collection and normalization strategies improve analytical reproducibility while reducing subjective interpretation of fluctuating wastewater signals. Integration of these environmental parameters into analytical and interpretive workflows enhances linkage between molecular measurements and epidemiologic context, improving confidence in surveillance outputs and supporting more consistent public health interpretation.

**Fig 2:**
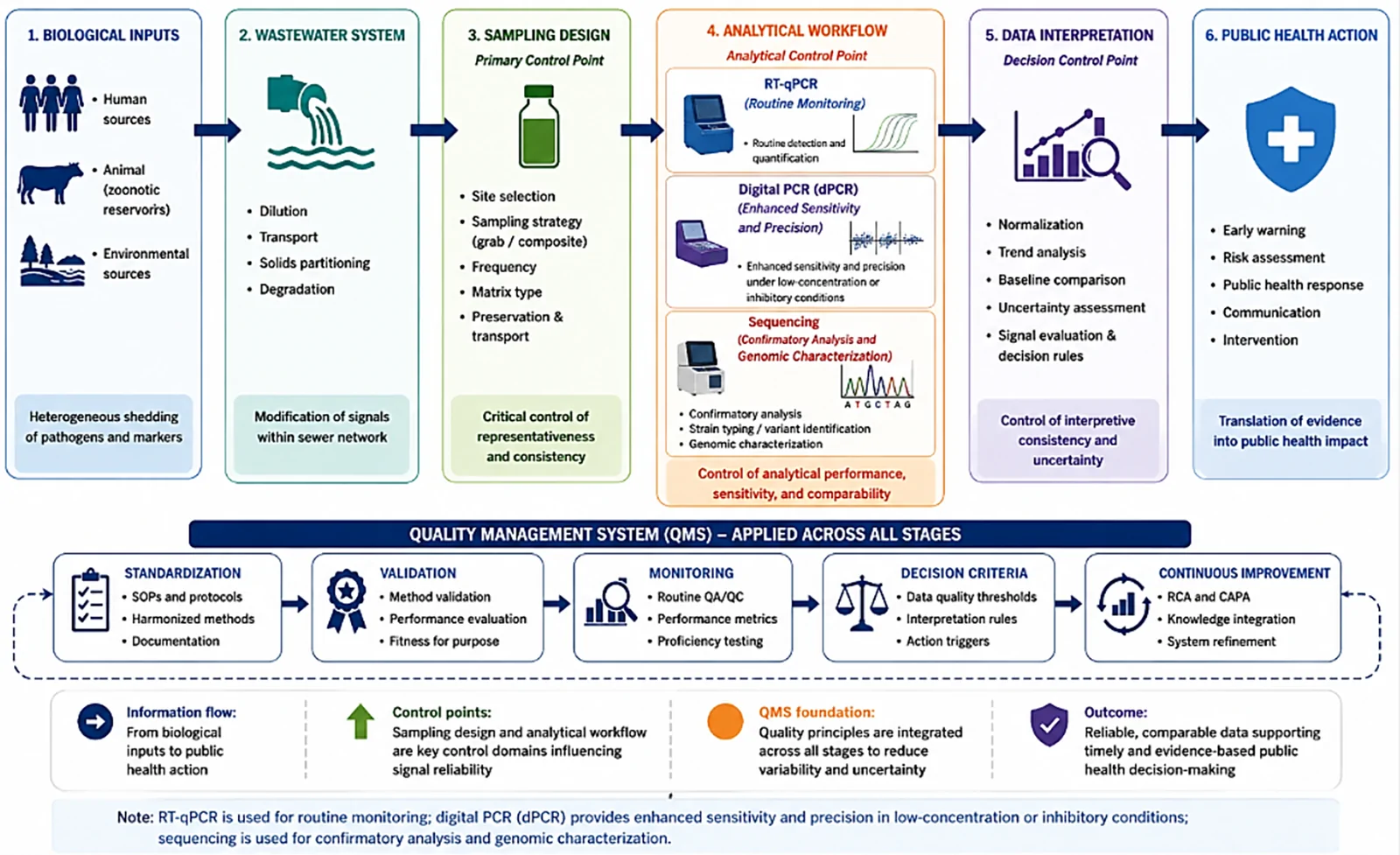
Conceptual framework of a QMS-driven, closed-loop surveillance architecture for zoonotic and emerging pathogens. The framework integrates strategic planning, risk assessment, operational surveillance processes, analytical interpretation, corrective and preventive actions (CAPA), and performance monitoring within an adaptive improvement cycle. Multi-source inputs—including clinical, wastewater, animal, environmental, and infrastructure-related data—are integrated to support standardized interpretation, uncertainty management, early warning, and coordinated public health decision-making. By incorporating feedback-driven quality management and structured escalation pathways, the framework supports resilient and interoperable surveillance across diverse public health settings.

As wastewater surveillance systems expand toward broader zoonotic and emerging pathogen applications, these analytical and quality-management components increasingly function within broader adaptive surveillance frameworks designed to support interoperability, uncertainty management, and coordinated public health response. Beyond operational standardization, wastewater surveillance for zoonotic and emerging pathogens requires adaptive architectures capable of managing uncertainty, heterogeneous inputs, and low-prevalence detection conditions. Figure 2 illustrates a conceptual QMS-driven, closed-loop surveillance architecture integrating strategic planning, risk assessment, analytical interpretation, corrective and preventive actions (CAPA), and continuous performance evaluation within an adaptive improvement cycle. The framework emphasizes interoperability across clinical, environmental, wastewater, and animal surveillance systems while supporting standardized interpretation, early warning, and coordinated public health decision-making under evolving outbreak conditions. By integrating feedback-driven quality management with structured analytical escalation pathways, this architecture extends wastewater surveillance from passive monitoring toward a resilient, decision-support surveillance system for emerging infectious disease preparedness.

## EPIDEMIOLOGIC INTERPRETATION AND GOVERNANCE IN WASTEWATER SURVEILLANCE

Wastewater surveillance is a powerful tool that provides both real-time and retrospective insights into the dynamics of infectious diseases. By analyzing time-resolved environmental samples, researchers can effectively track pathogen circulation, reconstruct transmission dynamics, identify early introductions, and characterize emerging or previously unrecognized pathogens (139–142). The detection of unexpected pathogens is primarily facilitated through advanced sequence-based approaches, particularly untargeted metagenomic sequencing, which surpasses the limitations of targeted molecular assays restricted to predefined agents. This capability is essential for addressing zoonotic and emerging pathogens, as it enables the detection of initial transmissions that may otherwise go unnoticed, ultimately enhancing public health responses and safeguarding communities.

The concentrations that are measured in various samples often reflect a complex interplay of factors, including heterogeneous shedding patterns, environmental degradation processes, hydraulic transport mechanisms, and the efficiency of analytical recovery methods (24, 143, 144). When considering the sources of these concentrations, it becomes evident that they typically arise from a mix of contributions from human activity, animal populations, and various environmental reservoirs. This mixed-source dynamic introduces additional layers of complexity, particularly for zoonotic pathogens. As a result, interpreting raw concentration data can be problematic and often unreliable unless thoroughly contextualized within relevant environmental and biological frameworks (69, 71, 145). This contextualization is crucial for accurately assessing the implications of these concentrations on public health and ecological stability.

Clear frameworks are essential for analyzing wastewater data. Key factors, such as water flow, rainfall, temperature, and the duration of water in the system, need to be included in the analysis. Viral load readings can be adjusted based on water flow, or the data can be separated into liquid and solid waste streams. Comparing current data to historical averages helps to spot trends over time and makes the results more stable. By treating these factors as measurable inputs rather than random uncertainties, wastewater monitoring can provide reliable and useful insights for public health agencies.

Longitudinal trend analysis improves the reliability of findings by examining temporal patterns at a single location. This approach minimizes variability across sites, making it easier to detect subtle signals from low-prevalence pathogens, early outbreaks, or re-emerging diseases (146). By utilizing stable baselines, rolling averages, and smoothed trends, this analysis enables the identification of significant deviations that reflect changing epidemiological dynamics.

The integration of complementary data streams, such as clinical case reports, hospitalization metrics, vaccination coverage data, syndromic surveillance information, and records from veterinary or environmental monitoring, significantly enhances confidence in interpreting health trends and patterns (15, 147). This multifaceted approach facilitates a more comprehensive understanding of disease dynamics, particularly for zoonotic pathogens—those capable of transmission between animals and humans. By distinguishing between contributions from human and animal sources, this cross-domain integration is vital for accurately tracking disease transmission pathways. Moreover, combining these diverse data sets improves our capacity to detect spillover events, in which pathogens jump from animals to humans. This proactive surveillance strategy is essential for preventing potential outbreaks and ensuring public health safety.

Finally, QMS principles extend to interpretation and decision-making. Predefined thresholds, structured reporting, and coordinated governance reduce variability, enhance transparency, and link analytical outputs to actionable public health decisions. Disciplined interpretation combines normalized signals, environmental context, and complementary epidemiologic data to ensure reliable, reproducible, and actionable insights, particularly for emerging and zoonotic pathogens.

## RETROSPECTIVE ARCHIVES, BIOBANKING, AND SURVEILLANCE PREPAREDNESS

Wastewater surveillance systems generate valuable retrospective records of pathogen circulation, enabling the reconstruction of transmission dynamics and the identification of emerging pathogens (139–142). These systems primarily use untargeted metagenomic sequencing to detect unexpected pathogens, which is particularly important for zoonotic and emerging infectious diseases that may go undetected in clinical data.

Archived wastewater samples can reveal pathogen presence before widespread clinical recognition (118, 140–142), aiding in distinguishing new introductions from previously undetected circulation (148). To realize their full potential, standardized preservation and metadata practices are essential. Nucleic acid extracts should be stored under appropriate cryogenic conditions to maintain integrity, while comprehensive documentation of sampling conditions and analytical methods is critical (22, 69).

Developing predefined biobanking frameworks with clear storage and reanalysis criteria can enhance the utility of archived materials. Trigger-based reanalysis, triggered by the detection of novel pathogens or unusual molecular patterns, improves operational efficiency. When supported by standardized preservation protocols and analytical frameworks, wastewater archives can strengthen early detection and response to evolving infectious disease threats.

## FUTURE DIRECTIONS AND EMERGING OPPORTUNITIES

Wastewater surveillance is evolving from targeted pathogen monitoring toward an integrated public health intelligence platform, with increasing relevance for zoonotic and emerging infectious diseases. A key future direction is the development of analytical, computational, and operational frameworks that enable timely and standardized interpretation across diverse pathogens, environments, and surveillance settings.

Future systems are expected to integrate automated sampling technologies, near-real-time analytical platforms, and centralized data infrastructures to support continuous monitoring and rapid signal interpretation. Standardized metadata collection, automated normalization processes, and predefined analytical thresholds linked to decision-support tools will further enhance interpretive consistency and responsiveness. Expanded use of sequencing approaches, including both targeted and untargeted methods, will improve detection of emerging and unexpected pathogens. Integration with complementary data streams—such as clinical, environmental, and veterinary surveillance—will strengthen a One Health approach to pathogen detection and response.

Advances in genomic technologies are central to this transformation. Improvements in sequencing depth, library preparation, and bioinformatic pipelines are enhancing the ability to detect and characterize mixed viral populations, including signals associated with zoonotic spillover and emerging variants (149–151). As these technologies mature, sequencing is likely to transition from a reactive escalation tool to a more routine component of surveillance, provided that performance standards and interpretive frameworks are established.

Emerging molecular detection platforms provide additional opportunities to improve sensitivity and timeliness. Approaches such as CRISPR-based detection and isothermal amplification offer rapid turnaround and high sensitivity, particularly for low-prevalence or newly emerging pathogens (152, 153). However, their application in complex wastewater matrices requires rigorous validation to ensure reproducibility and resistance to inhibition (22). Integration into standardized workflows will be essential to maintain comparability across surveillance systems.

Analytical modeling and data science are also expanding interpretive capabilities. Quantitative frameworks that incorporate pathogen-specific shedding dynamics, environmental transport processes, and uncertainty propagation can improve translation of wastewater concentrations into epidemiologically meaningful indicators (154). These approaches are particularly relevant for zoonotic pathogens, where multi-host transmission dynamics and environmental inputs complicate interpretation. Machine learning applied to harmonized data sets may further enhance anomaly detection and support early identification of emerging transmission patterns (155).

Infrastructure modernization represents another critical dimension of future development. Automated sampling systems, real-time metadata capture, and centralized data platforms can reduce operational variability and support coordinated analysis across jurisdictions (156, 157). Expansion of national and international data-sharing systems will improve cross-jurisdictional comparability and facilitate coordinated responses to emerging infectious disease threats (158, 159).

These technological and analytical advances are most effective when embedded in integrated One Health surveillance frameworks that link human, animal, and environmental monitoring. Such integration is essential for zoonotic pathogens, where early signals may arise at the interface of agricultural, environmental, and human systems. Coordinated surveillance across these domains can improve early detection of spillover events, enhance situational awareness, and strengthen preparedness.

Sustained progress will ultimately depend on maintaining methodological rigor and structured governance. Integration of emerging technologies must be accompanied by standardized quality frameworks, transparent reporting practices, and coordinated interpretive approaches. Continued investment in quality management systems, workforce development, and data infrastructure will be essential to ensure that technological innovation translates into reliable and actionable public health capabilities.

### Conclusion

Wastewater surveillance is increasingly evolving from a pathogen monitoring tool into an adaptive public health intelligence system capable of supporting early warning, situational awareness, and coordinated response to zoonotic and emerging infectious diseases. By providing population-level insights independent of clinical testing, wastewater surveillance offers important advantages for detecting emerging transmission patterns, particularly in settings where conventional surveillance may be limited, delayed, or fragmented.

The effectiveness of wastewater surveillance depends on its operation as an integrated and quality-managed system rather than a collection of isolated analytical measurements. Wastewater signals reflect the combined effects of heterogeneous pathogen shedding, environmental transformation, hydraulic variability, infrastructure characteristics, and analytical performance, requiring structured frameworks capable of accounting for system-level complexity and uncertainty. Framing surveillance within a QMS enables standardization across sampling, laboratory analysis, data interpretation, and reporting, thereby improving comparability, reproducibility, and evidence-based public health decision-making. Rather than treating uncertainty as an unavoidable limitation, QMS-driven surveillance architectures allow uncertainty to be systematically identified, evaluated, and incorporated into operational interpretation and response frameworks.

A central operational takeaway is that effective wastewater surveillance requires alignment among analytical methods, environmental context, infrastructure considerations, and epidemiologic interpretation. Tiered analytical strategies, standardized metadata integration, and predefined interpretive criteria enable surveillance systems to progress beyond descriptive monitoring toward actionable decision-support platforms. Integration with complementary surveillance systems and broader One Health frameworks further strengthens situational awareness across human, animal, and environmental domains while improving preparedness for emerging infectious disease threats.

An equally important priority is the integration of municipal wastewater and infrastructure authorities within QMS-aligned surveillance systems. Because sampling design directly influences the representativeness, stability, and interpretability of wastewater signals, sustained collaboration with municipal partners is essential to ensure appropriate site selection, standardized sampling practices, infrastructure awareness, and long-term operational reliability. Effective implementation further depends on coordinated governance across local, state, or regional, and federal levels. Local authorities support infrastructure management and surveillance execution, while state and regional systems facilitate data integration, standardization, and cross-jurisdictional coordination. At the national level, centralized frameworks support harmonized protocols, quality oversight, interoperable data systems, and aggregated situational awareness necessary for coordinated public health response.

Looking forward, the long-term impact of wastewater surveillance will depend not only on advances in molecular and sequencing technologies but also on their integration within disciplined operational, analytical, and governance frameworks. Continued investment in QMS implementation, workforce development, interoperable data infrastructure, and coordinated surveillance architectures will be essential to ensure that expanding analytical capabilities translate into reliable and actionable public health intelligence.

When implemented within a coordinated, quality-governed framework, wastewater surveillance can function as a resilient, interoperable, and scalable component of modern public health systems. By integrating environmental, clinical, veterinary, and infrastructure-level information into adaptive surveillance architectures, wastewater surveillance has the potential to support earlier detection, informed decision-making, and sustained preparedness for future zoonotic and emerging infectious disease threats.
